# Cancer models in preclinical research: A chronicle review of advancement in effective cancer research

**DOI:** 10.1002/ame2.12165

**Published:** 2021-03-29

**Authors:** Humna Sajjad, Saiqa Imtiaz, Tayyaba Noor, Yusra Hasan Siddiqui, Anila Sajjad, Muhammad Zia

**Affiliations:** ^1^ Department of Biotechnology Quaid‐i‐Azam University Islamabad Pakistan

**Keywords:** cancer cell lines, computational cancer models, genetically engineered mouse models, organoids, patient‐derived xenografts, personalized medicine

## Abstract

Cancer is a major stress for public well‐being and is the most dreadful disease. The models used in the discovery of cancer treatment are continuously changing and extending toward advanced preclinical studies. Cancer models are either naturally existing or artificially prepared experimental systems that show similar features with human tumors though the heterogeneous nature of the tumor is very familiar. The choice of the most fitting model to best reflect the given tumor system is one of the real difficulties for cancer examination. Therefore, vast studies have been conducted on the cancer models for developing a better understanding of cancer invasion, progression, and early detection. These models give an insight into cancer etiology, molecular basis, host tumor interaction, the role of microenvironment, and tumor heterogeneity in tumor metastasis. These models are also used to predict novel cancer markers, targeted therapies, and are extremely helpful in drug development. In this review, the potential of cancer models to be used as a platform for drug screening and therapeutic discoveries are highlighted. Although none of the cancer models is regarded as ideal because each is associated with essential caveats that restraint its application yet by bridging the gap between preliminary cancer research and translational medicine. However, they promise a brighter future for cancer treatment.

## Graphical abstract

In vitro and in vivo Animal models have been extensively used in cancer research. At present omics data and computational models are in practice. Each model has it own pros and cons.

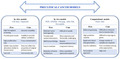

**Abbreviations**
CNA: copy number alteration
CRC: colorectal carcinoma
GEMMs: genetically engineered mouse models
HCC: hepatocellular carcinoma
KRAS: Kirsten rat sarcoma (viral oncogene homolog)
MMR: mismatch repair system
NGS: next‐generation sequencing
NRG: NOD rag gamma
NSG: nonobese diabetic (NOD)‐SCID gamma
OCM: oncopig cancer model
PDOs: patient‐derived organoids
PDX: patient‐derived xenograft
Rag: recombination‐activating gene
SCID: severe combined immunodeficiency syndrome
TP53: tumor protein 53

## 1 INTRODUCTION

Cancer is an epidemic disease causing approximately 8 million deaths annually all around the globe. Latest statistical data exhibit that human malignant growth is turning into the main source of death around the world. The absence of an intensive understanding of cancer biology in the recent era is a key hindrance to research the development, to understand the invasion, and to follow metastasis of cancer tumors.^1^, ^2^

Like other disease research, oncology research profoundly relies on a reliable and representative model framework. Nevertheless, we cannot define cancer as a single characterized tumor but instead a heterogeneous and immensely variable system. So that is why the choice of the most fitting model to best reflect the given tumor system is one of the real difficulties for cancer examination.^3^

Cancer models, either naturally found or artificially induced, have features in common with human cancers. The inability of in vitro cancer models to mimic the heterogeneity of human cancer cells, its microenvironment, and the stromal compartment has hindered the thorough understanding of tumor pathogenesis, therapeutic responses, and adverse reactions.^4^

Experimental systems for studying human cancer include cancer cell lines as well as 3D model organoids and organisms such as *Drosophila melanogaster,* zebrafish, and genetically engineered mouse model, pigs, patient‐derived xenografts (PDXs), and computational cancer models. These models form the basis to investigate cancer biochemical or genetic pathways and pathology. The cumulative information from cancer models helps in the understanding of the subtleties of cancer development in greater detail.^5^

**FIGURE 1:**
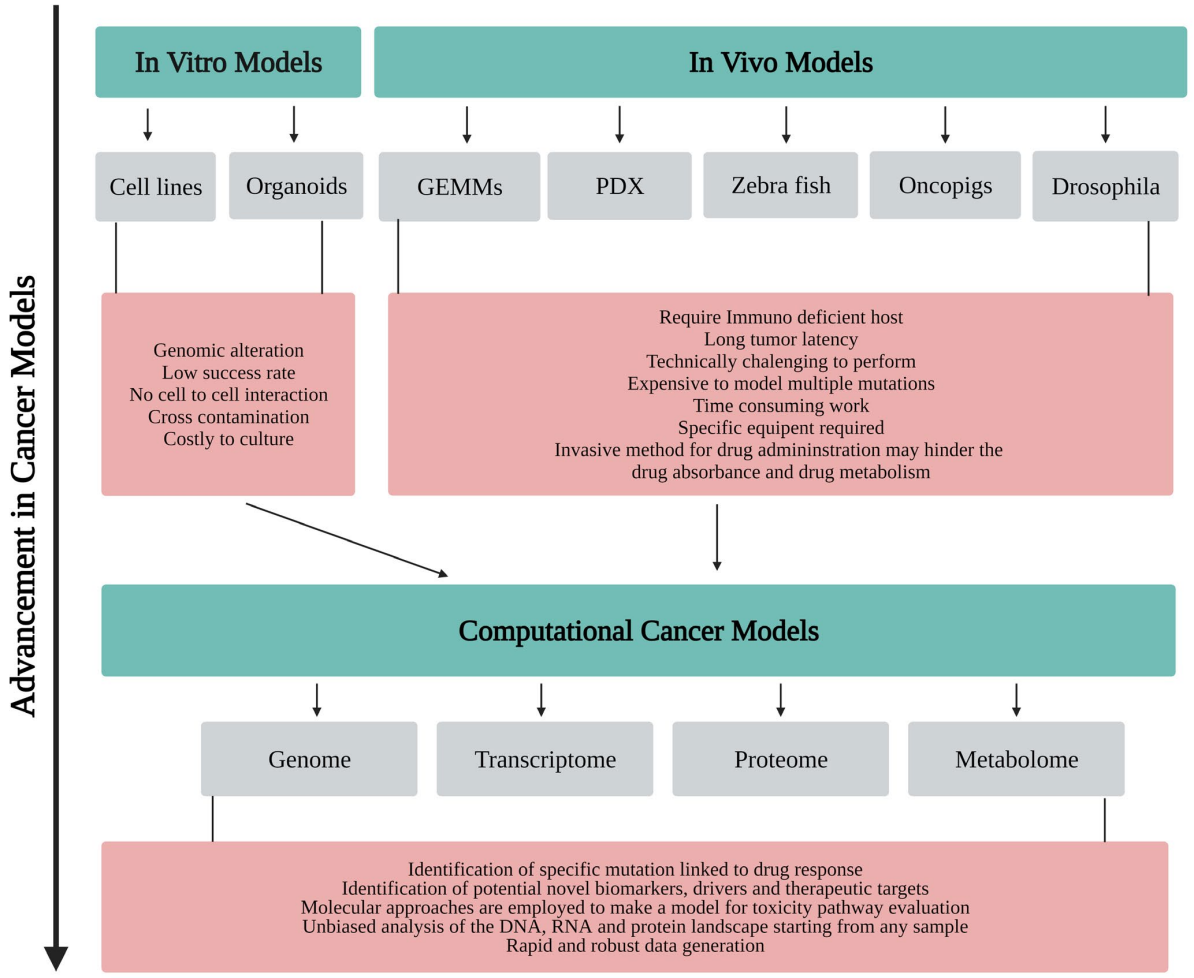
Advancement in cancer research models

To estimate clinical feedback in patients based on the model utilized, it is essential to get relatively a 50% hindrance in tumor development to accomplish a confirmed “response” to treatment and to utilize clinically applicable dosages of curative agents to observe survival. Moreover, it is essential to decide if tumor regeneration occurs when the medication is stopped, expecting this is the situation, whether the redevelopment is fast when treatment is delayed compared with before the treatment started. All cancer models aim to mimic at least some features of human cancer but in the end, we do not have a perfect model, yet it should be figured out how to intercept our information within the structure of the limitations of the test used.^6^ Figure 1 presents review flow chart.

## 2 CANCER CELL LINE

The cancer cell line is an in vitro tumor model that is regarded as a ubiquitous feature of oncology because it shows numerous intrinsic features of cancer and exhibits similar gene expression patterns.^7^, ^8^ Copy number alteration (CNA) and transcriptional profile of cancer cell lines show genomic alterations similar to primary human tumors. Cell lines are the foremost preclinical cancer model due to management ease, inexpensiveness, immortality, limited cellular heterogeneity, and high proliferation rates. Cancer cell lines are established by isolating cancer cells from patient‐specific organs and then growing in artificial culture media which on transplanting in immunocompetent mice led to the development of cancer cell line xenografts. Collections of cancer cell lines are the NCI‐60 cancer cell line panel, breast cancer cell line panel, and colorectal cancer (CRC) cell line panel.^9^ It has been confirmed by exome sequencing that primary tumor mutation in developed cell lines remains stable with extended passaging.^10^

Tumorgraft is developed by transplanting cancer cell lines in immunosuppressed mice and it serves as in vivo human cancer model. Extensive research conducted on a molecular and genomic comparison between patient tumor and tumor graft revealed that somatic and genomic mutation as well as gene expression patterns remain conserved between tumor and tumor grafts. As tissue physiology has a huge impact on tumor proliferation and drug pharmacokinetics, transplantation sites needed to be selected cautiously. A novel physiological cancer model is developed by orthotopic transplantation but it is restricted in certain tissues, especially when large cohorts of mice are under study. It has enhanced the clinical predictivity and almost every anticancer drug is evaluated in the human tumor xenograft model. Although in subcutaneous and renal capsule transplantation, tissue is easily assessed as each tumor cell evolves in specific microenvironment. On transplantation in non‐native microenvironment, cancer cell lines are unable to metastasize and give different response.^8^

Isogenic cancer cell lines are engineered by clustered regularly interspaced short palindromic repeats/CAS9 technology, which either removes or adds specific genes and serves as in vitro human cell line model.^11^ It assists in the analysis of specific mutations and thus used as a novel model for exploring targeted anticancer drug pharmacology.^12^ As the mismatch repair system (MMR) of DNA is responsible for maintaining genomic integration by recognizing mismatch even in the single base and short insertion‐deletion loops, cancer development begins when it becomes inactivated. Cancer cell lines rich in MMR and lacking MMR are being generated using isogenic cancer cell lines for comparison. MMR‐deficient cells develop resistance toward chemotherapeutics and show tolerance of DNA damage. Developing MMR‐deficient cancer cell line by isogenic cancer line help in finding compounds of rhodium metalloinsertor which efficiently hamper the outgrowth of MMR‐lacking cancer cells.^13^

Mislabeling, replacement of cell lines derived from different tissues, species, and individuals and their contamination with other cell lines is a serious quality control issue faced by the scientific community over 50 years. For example, eCv304 is claimed to be a spontaneously transformed human normal endothelial cell line but a later study shows that it is T24 bladder cancer cells18. It is still used as a model for endothelial cells in publications. The root cause of this grave issue is Cross‐contamination and mislabeling of the cell culture vessel during routine manipulation. Cell line verification by short tandem repeat profiling helps not only to increase the data credibility but also save money and effort used in mislabeled cell line identification.^14^

Cancer cell lines are not only highly amenable for in vitro and in vivo human cancer modeling but also carry some limitations. All tumor subtypes are not shown by existing cell lines; for example, luminal A breast cancer subtype is not found in the breast cancer cell line panel and exocrine‐like subtype is not found pancreatic ductal adenocarcinoma cell line panel. The high proliferation rate of cell lines tends towards discovering antiproliferative drugs thus negatively affecting the potential to identify broad‐spectrum anticancer compounds. Another caveat associated with the use of cancer cell lines is the lack of stromal components including lymphatic vessels, complex extracellular matrix, associated immune cells, blood, and fibroblasts.^8^ Prostate tumor is difficult to propagate in vitro. Variation in chromosome arrangements, differentiation markers, gene expression, karyotype, and growth rates of cell lines are observed on high passaging. Cancer cell lines are failed to precisely mimic the features of tumor cell growth in vivo due to the composition of culture media and fetal bovine serum presence in standard culture (Table 1).^15^

**TABLE 1:** Different cancer cell lines their derivation, tumor type, biological source, morphology, and growth mode

Cancer cell lines	Derived from	Tumor type	Biological source	Morphology	Growth mode	Ref.
Hela	*Homo sapiens*	Cervix adenocarcinoma	Human cervix	Epithelial	Adherent	16, 17
MCF‐7	*Homo sapiens*	Breast adenocarcinoma	Human breast (adenocarcinoma)	Epithelial‐like	Adherent	16, 18
HT‐29	*Homo sapiens*	Colon adenocarcinoma	Human colon	Epithelial	Adherent	16, 19
A549	*Homo sapiens*	Lung carcinoma	Human lung (carcinoma)	Epithelial	Adherent	16, 20
HEP‐G2	*Homo sapiens*	Hepatocellular carcinoma	Human liver	Epithelial	Adherent	16, 21
Cos7	*Cercopithecus aethiops*	SV‐40 transformed‐kidney	African green monkey kidney	Fibroblast	Adherent	16, 22
PC3	*Homo sapiens*	Prostate adenocarcinoma	Human prostate	Epithelial	Adherent	16, 23

## 3 PATIENT‐DERIVED XENOGRAFT

A xenograft is derived from the Greek word Xenos meaning foreign. It is obtained from one organism and implanted into other organisms. These implantations are mostly done in immunocompetent mice and are comprised of organs, tissue, or living cell. In the area of cancer research, xenografts are utilized to address key inquiries where it is important to rely on the utilization of animal models that show a close resemblance to the progression of the tumor in human patients.^24^

Xenograft models that contain primary carcinoma tissue is obtained from the patient's tumor tissue are built up at very low transit numbers; for example, less than 10 passages expelled from human patients to conserve the original or primary tumor characteristics.^25^ These characteristics include heterogeneity of cells, clinical biomolecular signatures, malignant genotypes and phenotypes, tumor structure, and vasculature.^24^ The basis for creating PDX models depends on the assumption that these PDX models will illustrate improved preclinical testing and are predictive of molecular cancer biology that is related to human cancer and how patients respond to cancer therapy.^26^ PDX models have been exhibited to be useful for (i) investigations of cancer metastasis and medication obstruction, (ii) personalized medication and treatment, and (iii) preclinical testing and discovery of new anticancer drug candidates.^27^

By surgery or biopsy methods, primary or metastatic tumors are collected and preserved as tissue structures.^26^ This resection of tumor specimens can develop gradually in immune‐deficient mice and instigate a switch toward applying patient‐derived tumor tissue xenograft models in the investigation of anticancer medications and treatment methods.^28^

The most widely recognized site of tumor implantation in mice is subcutaneous implantation (dorsal region), even though transplantation in a similar organ as the primary tumor might be an alternative and is known as orthotopic implantation; for example, pancreas, brain, oral cavity, ovary, breast, etc. Furthermore, many approaches and efforts have been made to implant tumor in the renal capsular site to increase the rate of engraftment. which has many advantages such as they can preserve histology of tumor tissue relative to the primary sample and progressive xenograft generations and can regenerate the original genotypic and phenotypic characteristics.^26^, ^29^ Furthermore, there are trial metastasis models in which controlled numbers of tumor cells are administered for metastasis, comparatively brief time is required for the advancement of metastasis, and metastases locales can then be determined.^30^

Advancement in cancer drug development has been hindered by an absence of preclinical cancer models that accurately evaluate clinical testing of significant novel compounds in human patients. So, these drawbacks have been overcome by the utilization of patient‐derived tumor xenograft in immunocompetent mice (preclinical models) such as nude mice, severe combined immunodeficiency mice (SCID), nonobese diabetic (NOD)‐SCID gamma mice, recombination‐activating gene (Rag), and NOD rag gamma mice.^24^ They are discussed in Table 2.

**TABLE 2:** Commonly utilized major immune‐compromised mouse strains and their advantages and disadvantages

Sr no	Advantages	Disadvantages	Ref.
Nu/nu (Nude mouse)	First immunodeficient mouse strain The total number of circulating lymphocytes is five to six times less in nude mice than in normal animals. The majority of these cells are B cells so they are used for numerous cancer metabolomics research Highly correct prediction rates in comparison to in vitro systems for resistance and sensitivity of a tumor	A significant limiting factor is the duration of testing because a time of at least 4 months is required for rapidly growing tumors and two years are required for slowly growing tumors after that test results can be obtained Nude mice are expensive they need special conditions behind laminar flow barriers to avoid infections	31, 32
Severe combined immunodeficiency syndrome (SCID)	No mature B and T cells and decreased NK activity Provide realistic heterogeneity of tumor cells It can predict the response of the drug of a tumor in human patients. It can allow the rapid analysis of human tumor response to a therapeutic regime	Since they are immunocompromised, they provide a less realistic tumor microenvironment They are expensive and technically complicated Low level of engraftment of human cells They have a very short life span of approximately 8.5 mo	34, 35
Nonobese diabetic (NOD)‐SCID gamma (NSG)	Easy to prepare NSG mice live longer than any other immune‐deficient mice Deficient in IL‐2 receptor gamma chain and lack of mature B, T, NK cells, and cytosolic signaling Used for metabolomics study for human immune deficiency virus	No primary immune response No multilineage hematopoiesis Expensive and technically complicated	36, 37
Recombination‐activating gene (Rag)	Similar to SCID mice possess RAG 1 or 2 mutations No mature B and T cells and radiation resistant	Surgical implantation is needed Human fetal tissue requirement Low or variable engraftment of human cells Might need additional conditioning to attenuate the primary immune response	36, 37, 39
NOD rag gamma (NRG)	It possesses RAG‐1 and IL‐2 receptor common gamma chain mutation NRG mice better tolerate irradiation allowing higher levels of human cord bloodstream cell engraftment than NSG mice NRG mice can prove useful for cell or tissue implantation studies	High engraftment levels of human cells in the newborn as compared to adults Xenospecific selection of human T cells might occur	36, 37, 40, 41

One basic part of huge preclinical investigations in PDX models is that these models help to organize potential clinical signs and are involved in the identification of potential drug efficacy biomarkers. In colorectal cancer, various examinations demonstrate that Kirsten rat sarcoma (KRAS)‐mutant PDX models do not react to cetuximab. KRAS wild‐type status is at present a well‐reported biomarker for this therapy in preclinical research. Almost similar observations were made for non‐small‐cell lung cancer. PDX models are additionally flexible instruments for reproducing resistance when used in treatment procedures utilized in the clinical experiment. This is particularly observed in the ovarian malignant tumor, in which when cisplatin is exposed, this results in the initiation of reluctance to cisplatin in a platinum‐sensitive model, exactly similar to that observed in the clinical setting. This model is used to investigate new factors, to choose medications to be tested in platinum‐safe patients.^42^ They are potentially powerful because they are generally biologically stable, and are indefinitely renewable. Breast cancer PDXs models recapitulate different aspects of the biology of the tumor and therefore they serve as an excellent model to carry out translational research.^43^

Patient‐derived xenografts, however, have limitations as well as having a different tumor microenvironment, are not amenable to genetic modification` and incorporation of immune system` since they are induced in immunodeficient mice so they do not recapitulate the commitment of the host immune system. The bacterial flora for carcinogenesis which is necessary for the early detection of cancer is not sustained in xenografts. They are not suitable for immunomodulatory testing for cancer prevention, initiation, and progression of genetics cancer modeling, along with low throughput drug screening. Their biobanking is not possible and they show genetic heterogeneity and epigenomic instability.^44^

## 4 GENETICALLY ENGINEERED MOUSE MODELS

Since the innate characteristics and physiology of xenografts do not outline the genetic characteristics of a human tumor, the genetically engineered mouse models (GEMMs) was established.^24^ Technical advancement over ongoing decades permits the investigators to make alterations in the genome of mice that conditionally or constitutively change the expression of important genes that led to the development of specific tumors. GEMMs have helped in oncogenesis to remove the molecular pathways and genome has been controlled in such a way to achieve loss or gain of oncogene or tumor suppressor gene function, the results of which are manifested in the phenotype of the tumor and have been helpful for therapy to validate key genes as targets.^45^, ^46^

The utilization of GEMMs for carcinogenicity evaluation started over 20 years ago.^24^ Currently, the European Commission thinks that the mouse seems to be a widely accepted animal for genetic modification to study the advanced drug therapeutics for curing various diseases for many reasons that are given below. First, the similarity of the mouse genome is 99% of the human genome. Second is the availability of a great molecular toolbox and their little size facilitates high‐throughput/large‐scale research making it a cost‐effective model. Transgenic adapted mice could give great preclinical safety testing and screening models for lead optimization and identification. The wide‐ranging phenotyping of GEMMs can give a better understanding of gene function that is related to human disease and health. In clinical research, the utilization of GEMM has proved itself effective in many cases such as the amount of medicine, method, or procedure for advanced treatment.^47^

In the review that follows, we give a historical viewpoint on the different types of GEMMs that are convenient for chemoprevention research. Transgenic mice or oncomice is the first GEMMs of human cancer. They give a direct method for surveying the results of the gain‐of‐function of specific genes for tumorigenesis. Among the second‐generation models, those following targeted deletion of Rb1 and Trp53, which build up a range of cancer growth phenotypes. It includes the targeted deletion of tumor suppressor genes in the germline. After the second generation, the next mouse model of human cancer was proposed. It includes Cre‐inducible gene targeting via Cre mouse alleles and Tet‐regulatable models which involves loss or gain of function. It controls the degree of targeting gene, temporal, and spatial. Tumor virus A model involves gain‐of‐function and include stochastic temporal and spatial regulation. RNAi gene silencing model which includes loss of function. The level of gene expression is controlled by the RNAi gene silencing model. This technology is still being developed.^45^, ^48^, ^49^ GEMMs are developed by altering the hereditary profile of the mice to an extent that genes involved in transformation are overexpressed, replaced, or deleted; eventually, the effect of transformation is examined after some time and therapeutic reactions to these tumors followed in vivo lead to the development of GEMMs.^48^

GEMMs of cancer must be evaluated carefully for their appropriateness to human disease and their predictive value for evaluating the prevention of cancer in humans. GEMMs of colon cancer can be utilized to investigate the impact of chemopreventive drugs on tumors established from genetic and epigenetic lesions that are related to multistage carcinogenesis. GEMMs of mammary cancer have shown that specific protective drugs might prevent the transformation from pre‐intrusive to obtrusive carcinoma. Wholesome enhancements that target various molecular pathways are effective in prostate cancer modeling. A definitive objective remains the generation of improved models that can be utilized for the predictive analysis of preventive reactions in humans.^46^

The main limitation of GEMMs is that it targets a few numbers of a gene which is normally not insightful of the complicated heterogeneity of human tumor cells. The establishment of GEMMs is expensive and tedious, frequently required long periods of work before validation. Tumor evolution in animals is variable and slow. They have different biochemistry, physiology, and anatomy compared to humans.

The critics of GEMMs proclaim that their significance for human cancer has not been developed. While the supporters of GEMMs claim that the issue is not that the models are not appropriate, yet that the experimental parameters have not been structured to successfully translate research from GEMMs to human cancer. Therefore, it is essential to develop criteria for evaluating the importance of a particular GEMMs for a given experimental framework. They include pathologic evaluation, disease progression, tumor microenvironment, molecular pathways, and environmental factors.^45^, ^50^, ^51^

In lung cancer, the precise mutations in Kras and P53 lead to the induction and development of tumors because of the activation of the NFκB pathway. From translational aspects, the discovery demonstrated that the use of antagonists to this pathway can help destroy cancerous cells. By using GEMMs, effective therapeutic preparations for various subtypes of different cancers and cancers with the same histological features can be developed.^52^ Hepatocellular carcinoma (HCC) has three subtypes each of which has almost similar survival but due to heterogeneous tumor properties but genetically engineered mice could be effective in the bench‐to‐beside research applications because of the possible genetic modifications. Also, in these GEMMs, a tetracycline‐dependent activation system is used to study the overexpression of *MYC* present in human carcinoma tumor. A relation between *E2f1* and *MYC* in tumorigenesis inside the liver has been reported by experimental analysis in mice by inducing genetic modifications.^53^

## 5 DROSOPHILA MELANOGASTER

*Drosophila melanogaster* has extensively contributed to elucidating the molecular basis of cancer biology by unveiling the action mechanism of proteins related to cancer. *D. melanogaster* is made as a cancer model by induction of mutation in larva using ethyl methanesulfonate (EMS). Tumorous tissue resides in the outer proliferative center (OPC) and central brain (CB) regions in the larval brain. The larval brain is then transplanted into the abdomens of adult female flies and transplanted tissue continuously proliferate in the abdomen, after several days the ovaries of the host are dissected and examined by immunofluorescence to detect labeled tumor cells that have metastasized. Engineered strains of *D. melanogaster* play a vital role in therapeutic drug discovery and provide a platform for drug screening. The level of detail and rate attained using *Drosophila* for genomic studies and functional assays are unparalleled to other mammalian cancer models. *D. melanogaster* gives insight into asymmetric division, centrosome dysfunction, genome instability, metabolism, and unscheduled gene expression which led to tumor initiation and cancer progression. The genome of *Drosophila* is homologous to the human as proteins that cause human cancer is found to be more than 50% orthologs between them.^54^ *Drosophila* has identified cell polarity mutants and their subsequent implication in human tumors.^55^

Recent flies’ cancer models include natural tumor, tumor‐induced by mutant obtained through genetic screening and tumor made à la carte. Testis and gut are more frequent natural tumors in wild‐type laboratory strains of *D. melanogaster*. Genetic screening analyses the entire genome of flies for identifying tumor suppressor genes and studying tumor suppressor function. Tumors made à la carte is designated as the third type of cancer model of *Drosophila* in which flies are designed to generate both the loss‐ and gain‐of‐function conditions that caused certain types of human cancers. The tumor is cooperatively produced by combining RAS activation and mitochondrial dysfunction which exerts an effect in neighboring cells and led them to exhibit metastatic behavior. Glioblastoma, gastric cancer, and rhabdomyosarcoma are à la carte designed cancer models. Different organs are being affected in larva and adult fly by recently developed tumors of *D. melanogaster*. Larval tumors show less similarity with human tumors while tumors of adult flies show distinct characteristics of malignancies such as they are neoplastic and immortal. Homologous genomic and centrosomal abnormalities are shown by *D. melanogaster* malignant neoplasms as observed in human cancer.^54^

The genetic mosaic technique helps to induce in living tissue somatic clones of mutant cells and permits the cells to have different genotypes to coexist in a single individual which helps to better understand the etiology of cancer and its progression. As multiple oncogenic mutations are sequentially acquired in subclones that caused tumorigenesis, conserved tumor suppressor genes and in vivo cancer progression are uncovered by generating clonal cell populations in *D. melanogaster*. It also has been shown in *Drosophila* using genetic mosaic screens that non‐cell‐autonomous tumor growth or progression is caused by many genes. For example, endocytic trafficking of transmembrane proteins is controlled by a component of the endosomal sorting complexes required for transport machinery, vacuolar protein sorting‐associated protein 25. Signaling pathways that are triggered by the transmembrane proteins get affected by the deregulation of such sorting systems causing tumorigenesis. Host tumor interaction is the contributing factor of cancer lethality, such as cachexia which is studied in *D. melanogaster* by allograft methods. Studies conducted on two independent flies applying the allograft method revealed that cancer cachexia by reinforcing insulin resistance in distant tissues systematically drive peripheral organ wasting.^56^

As dysfunctioning of the mechanisms responsible for genome stability led to structural alteration and point mutation, it contribute toward the development of malignant tumors. *Drosophila* larval brain tumors have first demonstrated the vital role played by failed asymmetric division for carcinogenesis. It has been shown that disturbing the division arrangement of *Drosophila*'s intestinal stem cells caused gut tumors.^54^, ^56^

Homologous recombination of flies is tiresome as compared to other model systems and flies cannot be kept frozen. As flies have only four chromosome pairs so aneuploidy is not an effective mode in them as only a few models with loss or gain of a single chromosome are possible.^54^, ^56^ In mammals before developing a secondary tumor and colonizing local tissue malignant cells that are undergoing metastasis enter the local blood vessel or lymph vessel, it is difficult to model in *Drosophila* as flies have rudimentary hematopoietic systems and different lymphatic system compared with mammals. Tumors induced in *Drosophila* have sufficiently reduced metastatic potential as compared to human tumors.^57^

## 6 PIG CANCER MODEL

The anatomical, physiological, and genetic similarities to humans, including chromosomal synteny and epigenetic homology, in addition to reduced cost for swine modeling, are striking advantages of this model so large‐sized animals efficiently represent the progression and development of cancer in humans.^58^ It has been effectively used for modeling leukemia, lymphoma, soft tissue sarcoma, pancreatic ductal adenocarcinoma, HCC, and other hematological cancers.^58^, ^59^ Phenotypic and genomic heterogeneity in pig herds is a result of their outbred nature, due to which genes that are crucial to cellular transformation are more observed in pigs. Chromosomal translocations that are commonly observed in cancer are effectively demonstrated in swine. The inducible nature of the Oncopig cancer model (OCM) makes it a perfect model for the identification of candidate biomarkers. TP53^R167H^ and KRAS^G12D^ are key tumor suppressors and oncogenes so a mutation in both assist OCM to effectively recapitulates transcriptional hallmarks of human disease while also exhibiting clinically relevant histologic and genotypic tumor phenotypes. OCM has the potential to develop various cancer types along with relevant comorbidities to circumvent the lethal impacts of comorbidities on healing strategies, patient management, and clinical outcomes. Pig models of heart failure, hypercholesterolemia, and hypertension are developed by using gene‐editing technology.^59^

Multiple genetically diverse lines of pigs with a high level of inbreeding have been found and used for cancer development; both preclinical research and simple technology are addressed by cultivation and characterization of these lines. Pig cancer model is developed by Cre recombinase bring site‐specific recombination between the LoxP recognition sites in fibroblast cell line taken from oncopig offspring and this led to the elimination of the Stop site and ultimately Lox‐Stop‐Lox sequence which prevents expression of oncogenes KRASG12D and TP53R167H get disturbed and show their expression on activation. Transgenic oncopig which develops from transgenic fibroblast cell lines is then infected with adenovirus encoding Cre recombinase (AdCre) which induced removal of the STOP codon allowing expression of both transgenes and tumors are induced at the site of injection in transgenic oncopig.^58^

For studying cancer in vivo, recently used porcine models are APC1311 porcine model of familial adenomatous polyposis, a heterozygous TP53 knockout model of spontaneous osteosarcomas, and a chemically precipitated porcine HCC model.^58^ Three essential kinds of cancer models in swine are spontaneous, induced, and genetically modified. Pigs' spontaneous cancer models are uncommon because pig's cancer develops with age and they do not have a long as they are used for the meal. Induced models give insight into the mechanism of tumorigenesis, for example, pigs can be induced to develop HCC by exposing them to *N*‐nitrosodiethylamine which resembles human HCC. Yorkshire pig line which naturally has SCID provides an opportunity to transplant human cancer and pancreatic carcinoma cells into them representing pigs as human tumor xenografts. The cancer xenograft model of pigs is developed by in utero cell transplantation. By casting off genes crucial for B‐cell and T‐cell development SCID swine model is developed which is referred to as “humanized pig” on engrafting with human immune cells and is used to analyze the role of the immune system in reaction to radiation and chemotherapy for the treatment of most cancers. Pigs are genetically modified by using transcription activator‐like effector nucleases or CRISPRs that induce efficient homologous recombination.^59^

The limitation of OCM is that they are unable to exhibit tumor‐stroma interaction and inefficient for the incorporation of the immune system, larger housing requirements compared to smaller animals, longer generation intervals, lower quality genome, and fewer genomic tools compared to mice and humans. When working with the pig, biosafety issues are more concerned since they are housed in pens.^60^

Genome sequencing with high‐throughput along with a collection of bioinformatics techniques precision‐genetic tools and gene expression profile or proteomics could be directly applied to these pig models. This model is not only a basic transitional and translational but also a transformational research tool for the examination of medicinal viability which tends to be used to direct correlative examinations for the progressively productive and predictable examination of new therapies. Its size permits the utilization of comparable systems and instruments that are utilized during clinical practice with regards to *Homo sapiens*, and the segmental idea of the liver of the pig empowers each Oncopig to manage its remedial control.^58^, ^60^

The porcine models have been produced for translational research in breast cancer, colorectal cancer, and pancreatic cancer.^61^ Spatially or temporarily controlled tumor induction along with the optimum dose administration, efficacy, and demonstration of toxicity can be exhibited by the OCM. About the fact that pigs have a longer life span as compared to mouse models, the OCM is not only a well‐established model but is also fail‐safe thus reducing the chances of error in translation and preclinical research experiments. The ability of the pig models to recapitulate the tumor microenvironment particularly in HCC has enabled the induction of multiple types of cancer including soft tissue sarcoma. The incidence of genetic mutations in pigs and humans occurs at a corresponding rate so heterogeneity can be assessed on new potential drugs.^62^

## 7 ORGANOID CANCER MODELING

The development of organoids as an ex vivo model system has revolutionized primary and clinical cancer studies during the last decade. Organoids are the infinitesimal of human organs and tissues, and functional features and architectures of a selected organ are efficiently represented. The organoid cancer model is developed by tumor cells isolated from tissue of cancer patient place in the extracellular matrix of specific culture media which develop it into cancer organoid.^4^ Organoids are amenable for molecular and cellular characterization and manipulation by various genetic tools and help in finding causative approaches in cancer etiology. New strategies to stratify cancer patients for both cytotoxic chemotherapies and targeted agents are provided by an early genome, transcriptome, and biochemical analyses of human cancer organoids. Organoids are matrix‐embedded cultures of primary epithelial cells that proliferate in a Wnt signaling and mitogen‐dependent manner continuously. On embedment of tissue‐derived stem cells into three‐dimensional matrix organoids as self‐sustainable structures are established.^63^

Not only do patient‐derived organoids (PDOs) show similar structural features with the primary tumor but also the expression pattern such as CNAs, transcriptional landscape, and mutation status of the tumor is maintained in PDOs. PDOs include HCC, breast, pancreatic, gastrointestinal, prostate, and bladder cancer. The PDOs usually lack critical elements such as immune cells, blood vessels, and different stromal cells. The major hindrance to the use of PDOs in cancer immunotherapy is the absence of immune components. As material exchange among cells pf PDOs is largely done through low rate infiltration rather than blood vessels which have lethal consequences on drug development and response.^64^

Some infectious pathogens such as in gallbladder carcinoma *Salmonella enterica,* in gastric cancer *Helicobacter* *pylori,* and Epstein‐Barr virus are essential risk factors for cancer development.^64^ Mechanism and relation between the infectious pathogen and cancer are studied by the co‐culture of organoids with specific pathogens. Studies on stomach organoids have illustrated the significant role played by chronic *H. pylori* infection in gastric cancer. *H. pylori* on microinjection provoke the strong primary inflammatory responses by locating and colonizing the gastric epithelium.^65^

Healthy organs are used for the growth of clonal organoid cultures and their genome sequencing facilitates the analysis of the mutation spectrum distinct to the organ. Intratumor heterogeneity is analyzed by developing clonal organoid cultures from distinct regions of the identical tumor. Organoid cultures are used for studying mutagenic processes due to genetic stability for a longer duration.^65^ Some of the cultural composition of common cancer organoids is shown in Table 3. Distinct lesions comparison from the same individual help to study processes involved in tumor evolution. Four independent matched sets of organoid cultures are generated from primary colorectal tumors and metastatic lesions isolated from the same patient, and exome sequencing confirmed that these lesions all evolved from a common origin and identified driver mutations shared among organoids derived from the same individual, suggesting that these driver mutations preceded metastatic dissemination.^64^

**TABLE 3:** Cultural composition of common cancer organoids

Organoids	Source	Extracellular matrix	Cultural components	Inhibitors	Cell types in organoid	Ref.
Stomach	hPSCs	Matrigel (growth factor reduced)	WNT, FGF, Noggin, Retinoic acid, EGF, ADMEM/F12, penicillin/streptomycin, l‐glutamine, B27, N2	A‐83‐01, Y27632	LGR5 + cells, mucous cells, gastric endocrine cells	66, 67
Prostate	hAdSc	Matrigel (growth factor reduced)	ADMEM, penicillin/streptomycin, primocin, GlutaMAX, B27, EGF, N‐acetylcysteine, FGF10, FGF‐basic, nicotinamide, testosterone, prostaglandin E_2_, Noggin, and R‐spondin	A‐83‐01, SB202190	Differentiated CK5 + basal and CK8 + luminal cells	66, 67
Pancrease	hAdSc	Matrigel	ADMEM/F12, penicillin/streptomycin, GlutaMAX, HEPES, B27, *N*‐acetylcysteine, EGF, R‐spondin‐1, gastrin 1, Wnt3A, Noggin, and FGF	A‐83‐01	Epithelial ductal cells	66, 67
Liver	hAdSc	Basement membrane extract	Activin A, Wnt,FGF,cAMP,glucocorticoids, ADMEM/F12, penicillin/streptomycin, GlutaMAX, HEPES, B27 (without vitamin A), N2, *N*‐acetylcysteine, nicotinamide, gastrin 1, EGF, FGF10, HGF, forskolin, R‐spondin‐1, Wnt3A, and Noggin	A‐83‐01, Y27632	Functional hepatocyte cells	66, 67

Organoids serve as a platform to study the tumor microenvironment. Signaling between malignant cells and the tumor microenvironment aids in generating a supportive niche for the tumor and also provides supportive therapeutic targets. Classical in vivo model systems are unable to locate the paracrine interactions within neoplasm cultures of cancer organoids and stromal cells are used to model the interactions between cancer cells and other cell types within the tumor microenvironment. An air‐liquid interface (ALI) system has been developed by Kuo and his colleagues in which epithelial organoids from resected human colorectal tumor tissue propagate in close association with αSMA‐positive myofibroblasts. Although it is not exposed to the selective pressure of monolayer culture fibroblasts and cancer cells remain unseparated, this model is unable for the functional dissection of cancer‐stromal signaling.^68^

Organoids can be engrafted into murine tissues to establish the organoid xenograft model that facilitates the in vivo analysis of human cancer biology such as breast and bladder cancer organoids introduced orthotopically for modeling each of these malignancies. In pancreatic cancer, orthotopically transplanted organoids can induce a microenvironment that is similar to primary human pancreatic cancer specimens than xenografts of monolayer cell lines. GEMMs of colon cancer tend to develop tumors in the small intestine, it puts limitations on its use for colon cancer modeling. To circumvent this limitation, organoids are orthotopically transplanted into the colon by transplantation into the murine cecum submucosa without damaging the colon. For this, first, the cecum is surgically exteriorized and collagen is used to hold the organoids at their position.^69^

Despite the tremendous revolution brought by organoids in cancer studies yet, they exhibit some limitations. As organoids are just an epithelial layer lacking an intrinsic microenvironment so they are considered imperfect reproductions. Further research is needed to be conducted on non‐epithelial organoids culture as in recent research organoids are derived mainly from the epithelium. Drug sensitivity, gene expression, and signaling pathways are severely impacted by growth stimulators and inhibitors.^70^ Further efforts are essential for the methodologies of organoid culturing and matrix formulations and organoids are not successfully generated from every specimen.^71^

## 8 ZEBRAFISH

A recent promising model to study human cancer is zebrafish (*Danio rerio*).^72^ A few normal human tumor types have been displayed in zebrafish utilizing transgenesis, affirming that the molecular mechanisms that support mammalian tumorigenesis likewise apply in zebrafish.^73^ Various factors including rapid development, chemical screening, amenable genetics, and its fitness for in vivo imaging make zebrafish an attractive model to cancer researchers.^74^ The zebrafish, by its forward genetics and vertebrate biology, has great potential as a cancer model system.^75^

Multiple approaches can be used to induce tumor formation in zebrafish including chemical carcinogenesis, mutant lines, xenotransplantation, and transgenic lines.^75^ Transplantation studies in zebrafish can be particularly effective in the study of tumor metastasis and its invasion. Zebrafish can be used in cancer studies either by the alternating nucleotide sequence of DNA, bioinformatics study of ‐omics data, examination of tumorigenesis, or by PDXs approach. Most of the studies on malignancy development in zebrafish originate from transgenic zebrafish that express oncogenes of mammals. The technique of transgenesis makes use of one of the advantages of zebrafish as a laboratory animal, that is, the ease of introducing the foreign DNA into cells of zebrafish and this DNA strand is expressed by injection into one‐cell embryos.^75^

Zebrafish has been indicated to be used as a model for studying unique human genetic disorders that are caused by a change in a single gene as well as the more pervasive human chromosomal abnormalities. The zebrafish can model a common human genetic disease involving multiple gene defects such as liver cancers. Zebrafish liver tumors have regular molecular similarities relating to the progressive condition of human liver tumors. It has been observed that there are 132 distinct genes in zebrafish liver tumors that showed strikingly comparable expression profiles relating to the progression of human liver cancer.^29^

The zebrafish cancer models are especially appropriate to predict novel cancer markers, differentiate between molecular prognostic biomarkers, and to establish their role in disease development. However, to develop different targeted therapies for particular oncogenes and signaling pathways, the zebrafish may form the basis to test and refine these targeted treatments in the preclinical period of development of drugs.^74^ Cancer‐causing medications on the zebrafish give advancement of induction of the disease and are much simpler to execute rather than mouse models.^75^

Zebrafish can spontaneously develop almost any type of tumor which has the same structure and comparatively similar signaling pathways as in humans. Their small size, large clutch size, low cost, the ability to generate hundreds of embryos from a single mating, translucent embryos, and ex utero growth of the embryo are the most important features that are extremely helpful in using zebrafish as a cancer model.^49^

Zebrafish models are manageable to genetic control. Forward genetics demonstrates its use in predicting cancer markers. Since the body of the non‐mammalian zebrafish is transparent so the tumor progression and cancer metastasis can be tracked efficiently. Thus, zebrafish serves as a reliable cancer model. Zebrafish have a small size which makes them simple and easy to house. Their zygotes are valuable in pharmacological research and drug screening.^74^

Difficulty in the examination of fixed tissue is the major disadvantage of zebrafish because sectioning embryos or larvae is tricky due to their small size.^76^ Also, there is relatively low tumor incidence, although these tumors are comparable in different mutants these tumors develop in life at a later stage.^72^ However, zebrafish is exceptionally fit to contribute insights in cancer biology and for providing a “whole‐organism test tube” for the rapid identification of the novel markers, to determine their functions, and the evaluation of their capacities, the investigation of host reactions, and development of anti‐cancer drugs.^74^

## 9 COMPUTATIONAL CANCER MODEL

Considering the certain risk factors (heterogeneity of tumor, the complexity of the disease, unsatisfactory clinical diagnosis of disease), genetic makeup, pharmacokinetic attributes, and other specific features distinct in each diseased individual would enable a personalized therapy approach to manage the severity of the tumor. In this regard, personalized medicine manifests the tailoring of a therapeutic approach to every patient with different genetic phenotypes and is assumed to become the model of future medical care. The most advanced research in system biology and the rapid progression of high‐throughput technologies, and similarly as the portrayal of various ‐omics, have significantly contributed to a switch in advanced medical and biological research from conventional hypothesis‐driven structures toward data‐driven studies and have encouraged the development of personalized or precision medications for more complex diseases, for example, cancer.^77^

Computational cancer model is a general term that includes computer‐dependent modeling linking to cancer treatment and tumor physiology.^78^ Computer‐based studies have been broadly applied to diagnose, observe, and predict cancer growth. For instance, via 3D microscopic imaging, tumor or tissue can be seen through computational simulation models. Numerical or computational models are most often related to algorithms and various computational software packages, so these models lack comparability and repeatability when contrasted with in vitro cellular cancer models.^79^

Presently, large‐scale computational models are being developed to study the signal transduction pathways in human cells. Researchers are now using an integrated programming platform PyBioS3 for the design, modeling, and simulation of different cell systems. The present model incorporates around 50 signaling pathways related to cancer and uses informative data based on the functional outcomes of genetic variations and mechanistic action of drugs.^80^, ^81^

To give a personalized prognosis, the models are individualized with the next‐generation sequencing (NGS) derived ‐omics data from the patient and the individual tumors (in case of cancer). It is analyzed for genetic alterations, for instance, single‐nucleotide polymorphisms, a fusion of genes, and gene mutation.^81^ Detailed information on ‐omics data is given in Table 4. Computational science of biology offers significant resources and efficient tools important for biological simulations, implementation of powerful cancer models by using significant experiment data, the progression of the disease, and strategic therapeutic evaluation. Computational and mathematical modeling has been used to understand the evolution of cancer. Computational tools provide the prospect of identifying new biomarkers in signaling cascades and auspicious targets for antitumor therapy. Cancer signaling network models have been established on time course experimental measuring protein expression and activity being utilized for the validation of simulation prediction and efficacy of drug target.^82^ To increase the translational achievement from cancer models to individuals, the results of experimental cancer models should compare to the predictions made by computational modeling. For example, the efficacy of drugs or medication on a computerized model of cell or animal model, primarily modifying this computerized model. This modification would then be adapted to each patient individually.^81^

**TABLE 4:** Advantages and disadvantages of ‐omics data

Omics data	Advantages	Disadvantages	Ref.
Genome	Identification of Single‐nucleotide Polymorphisms gives valuable data for early identification and prevention of various diseases	It is hard to predict the biological consequence of DNA by just genome examination due to epigenetics and post‐translational and transcriptional changes	83, 84
Transcriptome	Identification of the crucial pathways engaged in drug toxicity and response. Great reproducibility for laboratory studies	Insufficient information due to post‐translational changes	85
Proteome	Enable the examination of protein in complex systems. Reproducibility is increased by directly contrasting samples under the same electrophoretic conditions	Expensive and insensitive to low duplicated proteins not use for the whole proteome. Various outcomes because of post‐translational alteration	83, 86
Metabolome	Endogenic metabolites are less than genes, proteins, and transcripts, so less information is accessible to be interpreted. Identifying biomarkers of cancer research	Loss of various metabolites during tissue extraction. They are more dynamic and time‐sensitive	87

In the field of cancer research and therapy, computational systems also help in image study and its interpretation. Image assessment utilizing computerized tomography has been proposed recently for investigating individualized cancer responses.^81^

A very advanced computational model offers the opportunities for redesigning the experimental study, thus limiting the number of animal models required for experiments, significantly lessening the costs, and most importantly, improving the translational value of results produced. Computational models provide a better understanding of molecular alterations in disease‐related pathways and could give an efficient prescreening for the critical candidates’ selection. It can improve the knowledge of disease development and drug response.^81^

**FIGURE 2:**
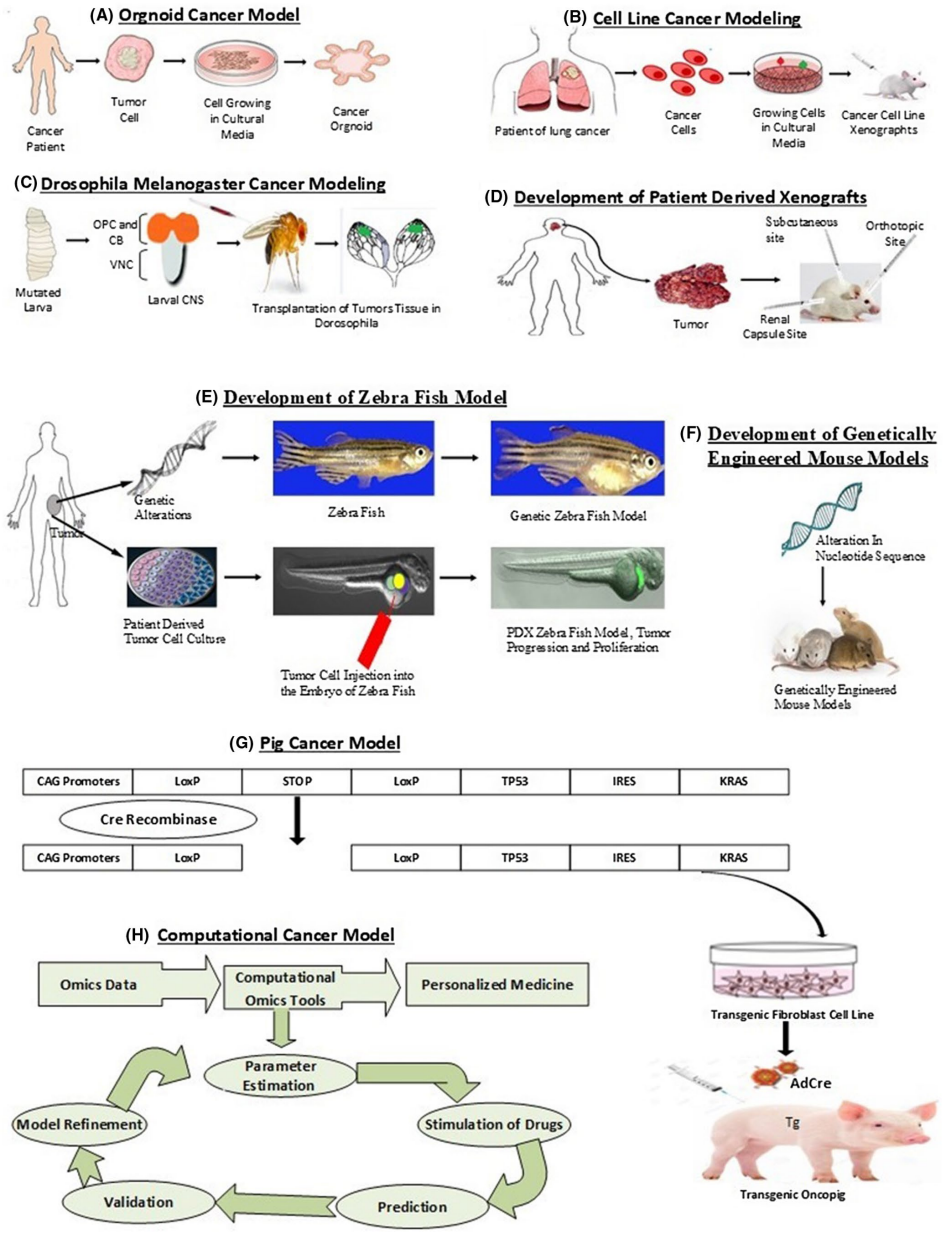
Development of cancer models. A, Representation of organoid cancer model development in artificial culture media by taking tumor cells from a cancer patient. B, Illustration of cancer cell lines grown in artificial culture media and its transplantation in immunocompetent mice. C, Mutated larval brain transplantation in the abdomen of female flies is shown to make the Drosophila Melanogaster model for cancer. D, Patient‐derived xenografts (PDXs) are developed by tumor cells that are derived from patients and transplanted into immunocompromised mice subcutaneously, orthotopically, or into the renal capsule. E, Zebrafish can be utilized in cancer studies either by the alternating nucleotide sequence of DNA or by the PDXs approach in which cancer tumor cells are developed from isolated or resected patient material and are introduced into larvae of zebrafish. F, Genetically Engineered Mouse Models are developed by altering the hereditary profile of the mice to an extent that genes involved in transformation are overexpressed, replaced, or deleted. G, Illustration of pig cancer model development is shown by infecting transgenic oncopig with AdCre to induce removal of STOP codon for expression of transgene and tumors at the site of injection. H, Computational cancer models are generated when omics data are generated from initial in vivo and in vitro experiments and are utilized to develop the process of computational tools. These tools involve the steps of parameter estimation, stimulation of drugs, prediction, validation, and model refinement

**FIGURE 3:**
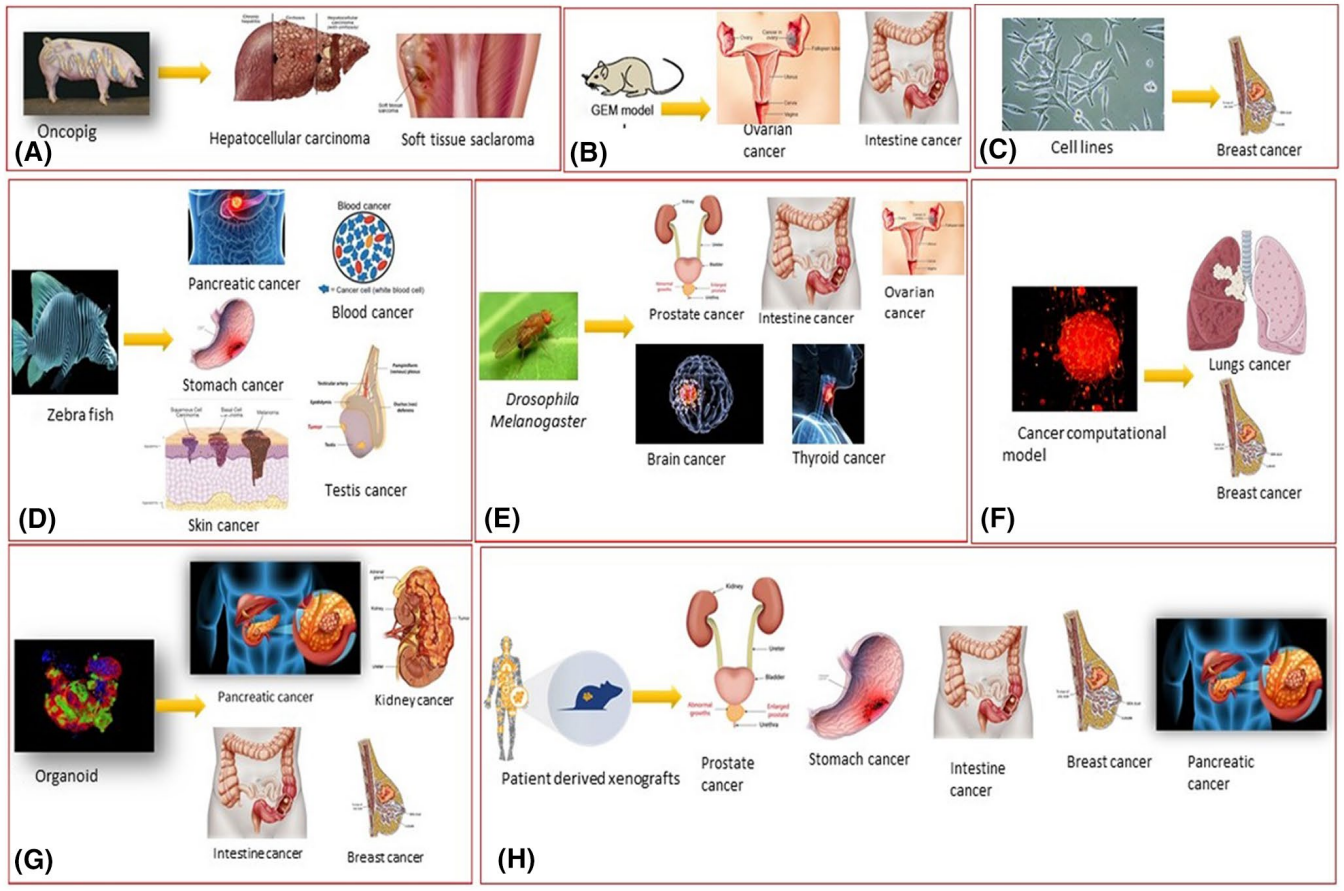
Application of cancer models in various cancer. Experimental models are being used to determine the characteristics of the different types of tumor proliferating in different organs inside the body. Despite the limitations and advantages of these models, each type of cancer growth associated with a particular organ (eg, lungs, breast, ovarian) interacts and responds to these experimental models differently. The following pictorial representation indicates the cancer model application and shows which experimental model depicts the properties of a specific cancer type more successfully than the other

It is obvious that currently working computational models do not reflect the entire complexity of simulated biological systems. An imperative boundary to the utilization of such models in a developmental and clinical context is the validity of prediction. A substitute strategy is to reduce the number of parameters by disentangling the model utilizing model reduction techniques ^81^, ^88^ The mechanism of the development of different cancer models is shown in Figures 2 and 3. In addition, their application area with their major strength and weaknesses are given in Table 5.

**TABLE 5:** Characteristics, strengths, and weaknesses of different cancer models

Cancer model	Classification based on modifications	Cancer type (application area)	Major strengths	Major weakness	References
Pig	APC1311 porcine model, heterozygous TP53 knockout pig model, porcine hepatocellular carcinoma (HCC) model	Adenomatous polyposis, spontaneous osteosarcomas, leukemia, lymphoma, soft tissue, pancreatic ductal adenocarcinoma, HCC	Reduced cost, efficient recapitulation of progression, and development of cancer, efficient representation of chromosomal translocation exhibiting clinically relevant histologic and genotypic tumor phenotypes	Unable to exhibit tumor‐stroma interaction, inefficient for the incorporation of the immune, larger housing requirements longer generation intervals, biosafety issues	5, 58, 60
Organoid	Prostate cancer organoids, pancreatic cancer organoids, Colorectal cancer organoids, Patient‐derived organoid	Prostate cancer, pancreatic cancer, colorectal cancer, breast cancer	Maintain the expression pattern such as copy number alterations (CNAs), transcriptional landscape, and mutation status of the tumor. Genetic stability for a longer duration was observed in the organoid cancer model. The tumor microenvironment can be studied	Patient‐derived organoid lacks immune components, Drug sensitivity, gene expression, and signaling pathways are severely impacted by growth stimulators and inhibitors	60, 63
Cancer cell line	K‐562, PC3, A375	Chronic myeloid leukemia, prostate adenocarcinoma, malignant melanoma	Management ease, inexpensiveness, immortality, limited cellular heterogeneity, high proliferation rates. Exhibits gene expression, patterns, and CNA similar to a human tumor	Cross‐contamination, mislabeling, high proliferation rate for antiproliferative drugs, lack of stromal components, Serial passage led to genotypic and phenotypic variation	11, 14
*Drosophila*	*Drosophila* NCOA4‐RET model, *Drosophila* CCDC6‐RET model	Multiple endocrine neoplasia Type 2	Gives insight into asymmetric division, centrosome dysfunction, genome instability, metabolism, and unscheduled gene expression. Genetic similarity with humans, centrosomal abnormalities similar to human and ease of maintenance and different lymphatic system compared to a human	Rudimentary hematopoietic systems and different lymphatic system and reduced the metastasis potential of the tumor compared to mammals	56, 57, 89
Genetically engineered mouse model (GEMM)	CB6F1‐rasH2 and B6.129‐Trp53tm1Brd mouse models	Lymphomas, osteosarcomas, and hemangiosarcoma, pancreatic cancer, breast cancer, and prostate cancer	Cost‐effectiveness with improved precision for early detection of the tumors on exposure to genotoxic and non‐genotoxic carcinogens, the tumor induction in several organ/tissues enables the target tissue evaluation with reduced possibility of false‐positive outcomes, mutated genes can be studied for driving the tumor‐initiating and signaling pathways	Only those tumors can be induced that are driven by specific signaling pathways, assessment of the drug safety and efficacy requires repeated dose‐response assessment, enhanced biosafety concerns because of the potential susceptibility to develop a tumor in shorter periods	90, 91
Patient‐derived xenograft (PDX)	Obtained from athymic nude mice (strain: Athymic nude—Foxn1^nu),^ NOD/severe combined immunodeficiency syndrome (SCID) mice (strain: NOD.CB17‐Prkdc^scid^/J), SCID/Bg mice (strain: CB17. Cg‐Prkdc^scid^Lyst^bg^‐J/Crl	Ovarian cancer, colorectal cancer, prostate cancer, Gastric cancer, Renal cell carcinoma, non‐small cell lung cancer	Heterogeneity of tumor is maintained so histological and molecular characterization is possible, personalized treatment with efficacious drug response tracking, the physiological, hormonal and oxygen conditions inpatient primary tumor originating site can be simulated from the expanded PDXs. Co‐clinical research, Drug screening, and biomarker, Precision medicine development	The metastatic implant is not possible, human tumor microenvironment interactions are not well established, development of PDX is a laborious and highly demanding step, engraftment failure, prolonged time for development and costly, immune‐modulating research is difficult when conventional PDX is used	43, 52, 93
Zebrafish	Fli1:EGFP zebrafish embryos, transgenic zebrafish Tg(fabp10:rtTA2s‐M2;TRE2:EGFP‐kras^G12V^)	HCC, lung adenocarcinoma, non‐small cell lung carcinoma, liver cancer, breast cancer, melanoma and squamous cell carcinoma (SCC), skin cancer	Characterization and visualization of the single cancerous cell, tumor detection, diagnosis followed by the preclinical investigation at an early stage, establishment of the testing system by positive control, tumorigenesis, and effect of inflammation are well demonstrated, cell imaging for high‐throughput drug testing and screening, genes involved in lineage‐specific pathways can be analyzed	Heterogeneity and tumor evolution can be detected because of the tumor transplant rejection, strategies employed for avoiding engraftment rejection may reduce the survival chances, specific equipment requirement, invasive methods for drug administration may hinder the drug absorbance, drug metabolism, and excretion are rarely exploited	94, 95
Computational cancer models	Genomics level, transcriptomics level, proteomics level, metabolomics level	Lung cancer, breast cancer, Pancreatic cancer	Unbiased analysis of the DNA, RNA, and protein landscape starting from any sample, Rapid and robust data generation, Creation of data repositories that can be used for other studies or validation by other researchers. Identification of potential novel biomarkers, drivers, and therapeutic targets, Identification of specific mutations linked to drug response. Molecular approaches are employed to make a model for toxicity pathway evaluation. Estimation of different chemical and physical properties of the molecules relevant to environmental fate and transport, Identification of cancer subtypes associated with particular cancers in different patients that in turn helped in the development of targeted therapeutics	High cost in terms of sample handling and starting amount, instrumentation, and time for data analysis and integration. The poor co‐relation between ‐omics approaches (eg, genomics, transcriptomics, proteomics, and metabolomics) Single‐cell analysis held great potential but is still underdeveloped. Tumors are heterogeneous and so ‐omics data from one part of the biopsy may not be representative of the whole tumor	98, 99

## 10 ROLE OF BIOTECHNOLOGY IN ONCOLOGY

Alteration in genome and protein regulation perturbs the normal mechanism of cell growth and causes the production of malignant cells. These molecular errors along with biomarkers, oncosuppressor genes, and related pathways are determined by using techniques of biotechnology such as in situ hybridization, cell culture, microarray analysis, and gene mapping.^102^ Cancer can be treated by monoclonal antibodies which hamper the functioning of the tumor cell by blocking receptors on the tumor surface.^103^

## 11 CONCLUSION

Eventually, the objective of any model is used to produce results that have a positive influence on patient wellbeing. The integrated utilization of in vitro, in vivo, and computational models in preclinical testing improves the cost efficacy of drug approval and development.

High proliferation rate and management ease of cancer cell lines give it an advantage over longer generation time and large housing requirements of the pig. Tumor graft effectively recapitulates the tumor in vivo which cell line is unable to mimic precisely. Studying tumor heterogeneity and microenvironment in organoids makes it a novel cancer model. Mouse models of human malignancy are significant tools for oncology research. PDXs are simple to use, reproducible, and moderately inexpensive. The shortcoming of PDX is that due to lack of intact immune system they do not recapitulate the genetics and histology of human tissue tumors as compared to GEMMs. That why this shortcoming is overcome by various GEMMs strains that are made to develop cancer that recapitulate the phenotypic, biochemical, and genetic features of specific human tumors. GEMMs hold significant promise for evaluating targeted therapeutics in the future. Although Drosophila has explicated the molecular basis of tumorigenesis due to having genomic similarity with humans as all other cancer models but structural and physiological differences with humans restricted its applications. This short come has been circumvented by oncopig which represents genomic, epigenomic, and chromosomal homology with humans. In contrast to traditional cell lines, zebrafish has many advantages. In the laboratory, zebrafish have been used for various purposes for the reason that it can give accurate results for different phenotypes but still, it has a lot of limitations. If accurate procedures and methods are used, zebrafish could be an excellent model for predicting novel biomarkers in the future.

Shortcomings of in vitro and in vivo models are overcome by computational models. Mechanistic computer models are established from the patient's ‐omics data and are developed for the analysis of drug effective responses and effects. But the existing computational model also does not fully represent the complexity of the human tumor being developed. Computational models provide better knowledge about molecular alterations in disease‐related pathways and could give a proficient prescreening for the critical patient selection. It can improve the understanding of disease progression and drug therapeutics response.

Cancer models, either in vitro, in vivo, or computational, enable us to conduct studies that are impractical on patients due to economic, moral, and welfare considerations. Gathering data and information from these models temporarily or permanently provide advantages to patients.

## References

[1] Wang C , Tang Z , Zhao Y , Yao R , Li L , Sun W . Three‐dimensional in vitro cancer models: a short review. Biofabrication. 2014;6(2):022001. 10.1088/1758-5082/6/2/022001 24727833

[2] Vargo‐Gogola T , Rosen JM . Modelling breast cancer: one size does not fit all. Nat Rev Cancer. 2007;7(9):659‐672. 17721431 10.1038/nrc2193

[3] Breitenbach M , Hoffmann J . Cancer models. Front Oncol. 2018;8:401. 30338241 10.3389/fonc.2018.00401 PMC6178941

[4] Voskoglou‐Nomikos T , Pater JL , Seymour L . Clinical predictive value of the in vitro cell line, human xenograft, and mouse allograft preclinical cancer models. Clin Cancer Res. 2003;9(11):4227‐4239. 14519650

[5] Schachtschneider KM , Schwind RM , Newson J , et al. The oncopig cancer model: an innovative large animal translational oncology platform. Front Oncol. 2017;7:190. 28879168 10.3389/fonc.2017.00190 PMC5572387

[6] Richmond A , Su Y . Mouse xenograft models vs GEM models for human cancer therapeutics. Disease Models & Mechanisms. 2008;1:78–82. 10.1242/dmm.000976 19048064 PMC2562196

[7] Domcke S , Sinha R , Levine DA , Sander C , Schultz N . Evaluating cell lines as tumour models by comparison of genomic profiles. Nat Commun. 2013;4(1):1‐10. 10.1038/ncomms3126 PMC3715866 23839242

[8] Goodspeed A , Heiser LM , Gray JW , Costello JC . Tumor‐derived cell lines as molecular models of cancer pharmacogenomics. Mol Cancer Res. 2016;14(1):3‐13. 26248648 10.1158/1541-7786.MCR-15-0189 PMC4828339

[9] Niu N , Wang L . In vitro human cell line models to predict clinical response to anticancer drugs. Pharmacogenomics. 2015;16(3):273‐285. 25712190 10.2217/pgs.14.170 PMC4358765

[10] Knudsen ES , Balaji U , Mannakee B , et al. Pancreatic cancer cell lines as patient‐derived avatars: genetic characterisation and functional utility. Gut. 2018;67(3):508‐520. 28073890 10.1136/gutjnl-2016-313133 PMC5868284

[11] Jin H , Cheng X , Nguyen N , et al. Using CRISPR/Cas9 to Generate Isogenic Cell Lines and Reference Standards for Applications in Cancer Diagnostics [abstract]. In: Proceedings of the American Association for Cancer Research Annual Meeting 2017; 2017 Apr 1‐5; Washington, DC, Philadelphia, PA: AACR; Cancer Res 2017;77(13 Suppl):Abstract nr 815. 10.1158/1538-7445.AM2017-815

[12] Haagensen EJ , Thomas HD , Mudd C , et al. Pre‐clinical use of isogenic cell lines and tumours in vitro and in vivo for predictive biomarker discovery; impact of KRAS and PI3KCA mutation status on MEK inhibitor activity is model dependent. Eur J Cancer. 2016;56:69‐76. 26820797 10.1016/j.ejca.2015.12.012

[13] Bailis JM , Gordon ML , Gurgel JL , Komor AC , Barton JK , Kirsch IR . An inducible, isogenic cancer cell line system for targeting the state of mismatch repair deficiency. PLoS One. 2013;8(10):e78726. 24205301 10.1371/journal.pone.0078726 PMC3812133

[14] Masters JR ; ASN‐ATCCSDOW . Cell line misidentification: the beginning of the end. Nat Rev Cancer. 2010;10(6):411–448. 10.1038/nrc2852 20448633

[15] Sharma SV , Haber DA , Settleman J . Cell line‐based platforms to evaluate the therapeutic efficacy of candidate anticancer agents. Nat Rev Cancer. 2010;10(4):241‐253. 20300105 10.1038/nrc2820

[16] Ferreira D , Adega F , Chaves R . The importance of cancer cell lines as in vitro models in cancer methylome analysis and anticancer drugs testing. In: Cesar LC , Elena AO , eds. Oncogenomics and cancer proteomics‐novel approaches in biomarkers discovery and therapeutic targets in cancer. 2013;139‐166.

[17] Li XM , Luo XG , He JF , et al. Induction of apoptosis in human cervical carcinoma HeLa cells by active compounds from Hypericum ascyron L. Oncol Lett. 2018;15(3):3944‐3950. 29556280 10.3892/ol.2018.7812 PMC5844083

[18] Vasyl'F C , Lukyanova NY , Kovalchuk O , Tryndyak VP , Pogribny IP . Epigenetic profiling of multidrug‐resistant human MCF‐7 breast adenocarcinoma cells reveals novel hyper‐and hypomethylated targets. Mol Cancer Ther. 2007;6(3):1089‐1098. 17363502 10.1158/1535-7163.MCT-06-0663

[19] Hu R , Kim BR , Chen C , Hebbar V , Kong A‐NT . The roles of JNK and apoptotic signaling pathways in PEITC‐mediated responses in human HT‐29 colon adenocarcinoma cells. Carcinogenesis. 2003;24(8):1361‐1367. 12819185 10.1093/carcin/bgg092

[20] Koparal AT , Zeytinoglu M . Effects of carvacrol on a human non‐small cell lung cancer (NSCLC) cell line, A549. Cytotechnology. 2003;43(1‐3):149‐154. 19003220 10.1023/B:CYTO.0000039917.60348.45 PMC3449592

[21] Du Q , Bian X‐L , Xu X‐L , Zhu B , Yu B , Zhai Q . Role of mitochondrial permeability transition in human hepatocellular carcinoma Hep‐G2 cell death induced by rhein. Fitoterapia. 2013;91:68‐73. 23994628 10.1016/j.fitote.2013.08.008

[22] Rommerskirch W , Graeber I , Grässmann M , Grässmann A . Homologous recombination of SV4O DNA in COS7 cells occurs with high frequency ma gene dose independent fashion. Nucleic Acids Res. 1988;16(3):941‐952. 2830596 10.1093/nar/16.3.941 PMC334729

[23] Tai S , Sun Y , Squires JM , et al. PC3 is a cell line characteristic of prostatic small cell carcinoma. Prostate. 2011;71(15):1668‐1679. 21432867 10.1002/pros.21383 PMC3426349

[24] Vandamme TF . Use of rodents as models of human diseases. J Pharm Bioallied Sci. 2014;6(1):2. 24459397 10.4103/0975-7406.124301 PMC3895289

[25] Tentler JJ , Tan AC , Weekes CD , et al. Patient‐derived tumour xenografts as models for oncology drug development. Nat Rev Clin Oncol. 2012;9(6):338‐350. 22508028 10.1038/nrclinonc.2012.61 PMC3928688

[26] Kopetz S , Lemos R , Powis G . The promise of patient‐derived xenografts: the best laid plans of mice and men. Clin Cancer Res. 2012;18(19):5160‐5162. 22912394 10.1158/1078-0432.CCR-12-2408 PMC4217576

[27] Jin K , Teng L , Shen Y , He K , Xu Z , Li G . Patient‐derived human tumour tissue xenografts in immunodeficient mice: a systematic review. Clin Transl Oncol. 2010;12(7):473‐480. 20615824 10.1007/s12094-010-0540-6

[28] Shimosato Y , Kameya T , Nagai K , et al. Transplantation of human tumors in nude mice. J Natl Cancer Inst. 1976;56(6):1251‐1260. 186624 10.1093/jnci/56.6.1251

[29] Cutz J‐C , Guan J , Bayani J , et al. Establishment in severe combined immunodeficiency mice of subrenal capsule xenografts and transplantable tumor lines from a variety of primary human lung cancers: potential models for studying tumor progression–related changes. Clin Cancer Res. 2006;12(13):4043‐4054. 16818704 10.1158/1078-0432.CCR-06-0252

[30] Wettersten HI , Ganti S , Weiss RH . Metabolomic profiling of tumor‐bearing mice. In: Methods in Enzymology. Vol. 543. Elsevier; 2014:275‐296. 24924138 10.1016/B978-0-12-801329-8.00014-3

[31] Loftus NJ , Lai L , Wilkinson RW , Odedra R , Wilson ID , Barnes AJ . Global metabolite profiling of human colorectal cancer xenografts in mice using HPLC–MS/MS. J Proteome Res. 2013;12(6):2980‐2986. 23631600 10.1021/pr400260h

[32] Pantelouris E . Athymic development in the mouse. Differentiation. 1973;1(6):437‐450. 4547146 10.1111/j.1432-0436.1973.tb00143.x

[33] Pantelouris E . Absence of thymus in a mouse mutant. Nature. 1968;217(5126):370‐371. 5639157 10.1038/217370a0

[34] Bosma GC , Custer RP , Bosma MJ . A severe combined immunodeficiency mutation in the mouse. Nature. 1983;301(5900):527‐530. 6823332 10.1038/301527a0

[35] Yano S , Nishioka Y , Izumi K , et al. Novel metastasis model of human lung cancer in SCID mice depleted of NK cells. Int J Cancer. 1996;67(2):211‐217. 8760590 10.1002/(SICI)1097-0215(19960717)67:2<211::AID-IJC11>3.0.CO;2-E

[36] Hogenes MC . B Cells and Regulatory T Cells in Graft Versus Host Disease: A Clinicopathological Study in Humanized Mice (Doctoral dissertation). Utrecht, Netherlands: Utrecht University; 2019.

[37] Akkina R . Human immune responses and potential for vaccine assessment in humanized mice. Curr Opin Immunol. 2013;25(3):403‐409. 23628166 10.1016/j.coi.2013.03.009 PMC3894824

[38] Shultz LD , Lyons BL , Burzenski LM , et al. Human lymphoid and myeloid cell development in NOD/LtSz‐scid IL2Rγnull mice engrafted with mobilized human hemopoietic stem cells. J Immunol. 2005;174(10):6477‐6489. 15879151 10.4049/jimmunol.174.10.6477

[39] Mombaerts P , Iacomini J , Johnson RS , Herrup K , Tonegawa S , Papaioannou VE . RAG‐1‐deficient mice have no mature B and T lymphocytes. Cell. 1992;68(5):869‐877. 1547488 10.1016/0092-8674(92)90030-g

[40] Hülsdünker J , Zeiser R . Insights into the pathogenesis of GvHD: what mice can teach us about man. Tissue Antigens. 2015;85(1):2‐9. 25532439 10.1111/tan.12497

[41] Pearson T , Shultz LD , Miller D , et al. Non‐obese diabetic–recombination activating gene‐1 (NOD–Rag 1 null) interleukin (IL)‐2 receptor common gamma chain (IL 2 rγnull) null mice: a radioresistant model for human lymphohaematopoietic engraftment. Clin Exp Immunol. 2008;154(2):270‐284. 18785974 10.1111/j.1365-2249.2008.03753.x PMC2612717

[42] Hidalgo M , Amant F , Biankin AV , et al. Patient‐derived xenograft models: an emerging platform for translational cancer research. Cancer Discovery. 2014;4(9):998‐1013. 25185190 10.1158/2159-8290.CD-14-0001 PMC4167608

[43] Dobrolecki LE , Airhart SD , Alferez DG , et al. Patient‐derived xenograft (PDX) models in basic and translational breast cancer research. Cancer Metastasis Rev. 2016;35(4):547‐573. 28025748 10.1007/s10555-016-9653-x PMC5396460

[44] Pillai S , Uthamanthil R . PDX models: History and development. In: Rajesh U , Peggy T , eds. Patient Derived Tumor Xenograft Models. London: Elsevier; 2017:1‐12.

[45] Abate‐Shen C , Brown PH , Colburn NH , et al. The untapped potential of genetically engineered mouse models in chemoprevention research: opportunities and challenges. Cancer Prev Res. 2008;1(3):161‐166. 10.1158/1940-6207.CAPR-08-0076 PMC2758098 19138951

[46] Green JE , Hudson T . The promise of genetically engineered mice for cancer prevention studies. Nat Rev Cancer. 2005;5(3):184‐198. 15738982 10.1038/nrc1565

[47] Brandon‐Warner E , Schrum LW , Schmidt CM , McKillop IH . Rodent models of alcoholic liver disease: of mice and men. Alcohol. 2012;46(8):715‐725. 22960051 10.1016/j.alcohol.2012.08.004 PMC3496818

[48] Hanahan D , Wagner EF , Palmiter RD . The origins of oncomice: a history of the first transgenic mice genetically engineered to develop cancer. Genes Dev. 2007;21(18):2258‐2270. 17875663 10.1101/gad.1583307

[49] Van Dyke T , Jacks T . Cancer modeling in the modern era: progress and challenges. Cell. 2002;108(2):135‐144. 11832204 10.1016/s0092-8674(02)00621-9

[50] Frese KK , Tuveson DA . Maximizing mouse cancer models. Nat Rev Cancer. 2007;7(9):654‐658. 10.1038/nrc2192 17687385

[51] Olive KP , Tuveson DA . The use of targeted mouse models for preclinical testing of novel cancer therapeutics. Clin Cancer Res. 2006;12(18):5277‐5287. 17000660 10.1158/1078-0432.CCR-06-0436

[52] Porru M , Leonetti C . The role of mouse models in translational cancer research: present and future directions. Transl Med Rep. 2018;2(1):64–69.

[53] Brown ZJ , Heinrich B , Greten TF . Mouse models of hepatocellular carcinoma: an overview and highlights for immunotherapy research. Nat Rev Gastroenterol Hepatol. 2018;15(9):536‐554. 29904153 10.1038/s41575-018-0033-6

[54] Gonzalez C . Drosophila melanogaster: a model and a tool to investigate malignancy and identify new therapeutics. Nat Rev Cancer. 2013;13(3):172‐183. 23388617 10.1038/nrc3461

[55] Enomoto M , Siow C , Igaki T . Drosophila as a cancer model. In: Masamitsu Y , ed. Drosophila Models for Human Diseases. Cham, Switzerland: Springer; 2018:173‐194. 10.1007/978-981-13-0529-0_10 29951820

[56] Rudrapatna VA , Cagan RL , Das TK . Drosophila cancer models. Dev Dyn. 2012;241(1):107‐118. 22038952 10.1002/dvdy.22771 PMC3677821

[57] Miles WO , Dyson NJ , Walker JA . Modeling tumor invasion and metastasis in Drosophila. Dis Models Mech. 2011;4(6):753‐761. 10.1242/dmm.006908 PMC3209645 21979943

[58] Schook LB , Collares TV , Hu W , et al. A genetic porcine model of cancer. PLoS One. 2015;10(7):e0128864. 26132737 10.1371/journal.pone.0128864 PMC4488487

[59] Watson AL , Carlson DF , Largaespada DA , Hackett PB , Fahrenkrug SC . Engineered swine models of cancer. Front Genet. 2016;7:78. 27242889 10.3389/fgene.2016.00078 PMC4860525

[60] Bailey KL , Carlson MA . Porcine models of pancreatic cancer. Front Oncol. 2019;9:144. 30915276 10.3389/fonc.2019.00144 PMC6423062

[61] Kalla D , Kind A , Schnieke A . Genetically engineered pigs to study cancer. Int J Mol Sci. 2020;21(2):488. 10.3390/ijms21020488 PMC7013672 31940967

[62] Robertson N , Schook LB , Schachtschneider KM . Porcine cancer models: potential tools to enhance cancer drug trials. Expert Opin Drug Discovery. 2020;15(8):1‐10. 10.1080/17460441.2020.1757644 32378979

[63] Sachs N , Clevers H . Organoid cultures for the analysis of cancer phenotypes. Curr Opin Genet Dev. 2014;24:68‐73. 24657539 10.1016/j.gde.2013.11.012

[64] Huch M , Knoblich JA , Lutolf MP , Martinez‐Arias A . The hope and the hype of organoid research. Development. 2017;144(6):938‐941. 28292837 10.1242/dev.150201

[65] Yang H , Sun L , Liu M , Mao Y . Patient‐Derived Organoids: a Promising Model for Personalized Cancer Treatment. Oxford: Oxford University Press; 2018. 10.1093/gastro/goy040 PMC6225812 30430011

[66] Xu H , Lyu X , Yi M , Zhao W , Song Y , Wu K . Organoid technology and applications in cancer research. J Hematol Oncol. 2018;11(1):116. 30219074 10.1186/s13045-018-0662-9 PMC6139148

[67] Drost J , Clevers H . Organoids in cancer research. Nat Rev Cancer. 2018;18(7):407‐418. 29692415 10.1038/s41568-018-0007-6

[68] Tuveson D , Clevers H . Cancer modeling meets human organoid technology. Science. 2019;364(6444):952‐955. 31171691 10.1126/science.aaw6985

[69] Kim J , Koo B‐K , Knoblich JA . Human organoids: model systems for human biology and medicine. Nat Rev Mol Cell Biol. 2020;21(10):571‐584. 32636524 10.1038/s41580-020-0259-3 PMC7339799

[70] Lo Y‐H , Karlsson K , Kuo CJ . Applications of organoids for cancer biology and precision medicine. Nat Cancer. 2020;1(8):761‐773. 10.1038/s43018-020-0102-y PMC8208643 34142093

[71] Pauli C , Hopkins BD , Prandi D , et al. Personalized in vitro and in vivo cancer models to guide precision medicine. Cancer Discovery. 2017;7(5):462‐477. 28331002 10.1158/2159-8290.CD-16-1154 PMC5413423

[72] Stoletov K , Klemke R . Catch of the day: zebrafish as a human cancer model. Oncogene. 2008;27(33):4509‐4520. 18372910 10.1038/onc.2008.95

[73] Lieschke GJ , Currie PD . Animal models of human disease: zebrafish swim into view. Nat Rev Genet. 2007;8(5):353‐367. 17440532 10.1038/nrg2091

[74] Mione MC , Trede NS . The zebrafish as a model for cancer. Dis Models Mech. 2010;3(9‐10):517‐523. 10.1242/dmm.004747 PMC2931530 20354112

[75] Amatruda JF , Shepard JL , Stern HM , Zon LI . Zebrafish as a cancer model system. Cancer Cell. 2002;1(3):229‐231. 12086858 10.1016/s1535-6108(02)00052-1

[76] Brown HK , Schiavone K , Tazzyman S , Heymann D , Chico TJ . Zebrafish xenograft models of cancer and metastasis for drug discovery. Expert Opin Drug Discovery. 2017;12(4):379‐389. 10.1080/17460441.2017.1297416 28277839

[77] Savoia C , Volpe M , Grassi G , Borghi C , Agabiti Rosei E , Touyz RM . Personalized medicine—a modern approach for the diagnosis and management of hypertension. Clin Sci. 2017;131(22):2671‐2685. 10.1042/CS20160407 PMC5736921 29109301

[78] Barbolosi D , Ciccolini J , Lacarelle B , Barlési F , André N . Computational oncology—mathematical modelling of drug regimens for precision medicine. Nat Rev Clin Oncol. 2016;13(4):242. 26598946 10.1038/nrclinonc.2015.204

[79] Morgan MM , Johnson BP , Livingston MK , et al. Personalized in vitro cancer models to predict therapeutic response: challenges and a framework for improvement. Pharmacol Ther. 2016;165:79‐92. 27218886 10.1016/j.pharmthera.2016.05.007 PMC5439438

[80] Wierling C , Kessler T , Ogilvie LA , Lange BM , Yaspo M‐L , Lehrach H . Network and systems biology: essential steps in virtualising drug discovery and development. Drug Discovery Today Technol. 2015;15:33‐40. 10.1016/j.ddtec.2015.07.002 26464088

[81] Ogilvie LA , Kovachev A , Wierling C , Lange BM , Lehrach H . Models of models: a translational route for cancer treatment and drug development. Front Oncol. 2017;7:219. 28971064 10.3389/fonc.2017.00219 PMC5609574

[82] Jean‐Quartier C , Jeanquartier F , Jurisica I , Holzinger A . In silico cancer research towards 3R. BMC Cancer. 2018;18(1):408. 29649981 10.1186/s12885-018-4302-0 PMC5897933

[83] Karahalil B . Overview of systems biology and omics technologies. Curr Med Chem. 2016;23(37):4221‐4230. 27686657 10.2174/0929867323666160926150617

[84] Jiang J , Wolters JE , van Breda SG , Kleinjans JC , de Kok TM . Development of novel tools for the in vitro investigation of drug‐induced liver injury. Expert Opin Drug Metab Toxicol. 2015;11(10):1523‐1537. 26155718 10.1517/17425255.2015.1065814

[85] Wang Z , Gerstein M , Snyder M . RNA‐Seq: a revolutionary tool for transcriptomics. Nat Rev Genet. 2009;10(1):57‐63. 19015660 10.1038/nrg2484 PMC2949280

[86] Wright P , Noirel J , Ow S‐Y , Fazeli A . A review of current proteomics technologies with a survey on their widespread use in reproductive biology investigations. Theriogenology. 2012;77(4):738‐765.e52. 22325247 10.1016/j.theriogenology.2011.11.012

[87] Monteiro M , Carvalho M , Bastos M , Guedes de Pinho P . Metabolomics analysis for biomarker discovery: advances and challenges. Curr Med Chem. 2013;20(2):257‐271. 23210853 10.2174/092986713804806621

[88] Jones W , Alasoo K , Fishman D , Parts L . Computational biology: deep learning. Emerging Top Life Sci. 2017;1(3):257‐274. 10.1042/ETLS20160025 PMC7289034 33525807

[89] Levinson S , Cagan RL . Drosophila cancer models identify functional differences between ret fusions. Cell Rep. 2016;16(11):3052‐3061. 27626672 10.1016/j.celrep.2016.08.019 PMC5858711

[90] Webster JD , Santagostino SF , Foreman O . Applications and considerations for the use of genetically engineered mouse models in drug development. Cell Tissue Res. 2020;380(2):325‐340. 31486957 10.1007/s00441-019-03101-y

[91] Weng C‐C , Lin Y‐C , Cheng K‐H . The use of genetically engineered mouse models for studying the function of mutated driver genes in pancreatic cancer. J Clin Med. 2019;8(9):1369. 10.3390/jcm8091369 PMC6780401 31480737

[92] Annunziato S , Lutz C , Henneman L , et al. In situ CRISPR‐Cas9 base editing for the development of genetically engineered mouse models of breast cancer. EMBO J. 2020;39(5):e102169. 31930530 10.15252/embj.2019102169 PMC7049816

[93] Jung J , Seol HS , Chang S . The generation and application of patient‐derived xenograft model for cancer research. Cancer Res Treat. 2018;50(1):1. 28903551 10.4143/crt.2017.307 PMC5784646

[94] Liu C , Zhang Y , Lim S , et al. A zebrafish model discovers a novel mechanism of stromal fibroblast‐mediated cancer metastasis. Clin Cancer Res. 2017;23(16):4769‐4779. 28420724 10.1158/1078-0432.CCR-17-0101

[95] Yang Q , Salim L , Yan C , Gong Z . Rapid analysis of effects of environmental toxicants on tumorigenesis and inflammation using a transgenic zebrafish model for liver cancer. Mar Biotechnol. 2019;21(3):396‐405. 10.1007/s10126-019-09889-8 30852708

[96] Bootorabi F , Manouchehri H , Changizi R , et al. Zebrafish as a model organism for the development of drugs for skin cancer. Int J Mol Sci. 2017;18(7):1550. 10.3390/ijms18071550 PMC5536038 28718799

[97] Letrado P , de Miguel I , Lamberto I , Díez‐Martínez R , Oyarzabal J . Zebrafish: speeding up the cancer drug discovery process. Can Res. 2018;78(21):6048‐6058. 10.1158/0008-5472.CAN-18-1029 30327381

[98] Parsons J , Francavilla C . ‘Omics approaches to explore the breast cancer landscape. Front Cell Dev Biol. 2019;7:395. 32039208 10.3389/fcell.2019.00395 PMC6987401

[99] Saeidnia S , Manayi A , Abdollahi M . The pros and cons of the in‐silico pharmaco‐toxicology in drug discovery and development. Int J Pharmacol. 2013;9(3):176‐181.

[100] Bast F . Cancer Phylogenetics: Computational Modeling of Tumor Evolution. Bathinda, India: Nova Science Publishers, Inc.; 2012.

[101] Mathew JP , Taylor BS , Bader GD , et al. From bytes to bedside: data integration and computational biology for translational cancer research. PLoS Comput Biol. 2007;3(2):e12. 17319736 10.1371/journal.pcbi.0030012 PMC1808026

[102] Francia G , Kerbel RS . Raising the bar for cancer therapy models. Nat Biotechnol. 2010;28(6):561‐562. 20531333 10.1038/nbt0610-561

[103] Scott AM , Allison JP , Wolchok JD . Monoclonal antibodies in cancer therapy. Cancer Immunity Archive. 2012;12(1).6–14. PMC3380347 22896759

